# Supplementary material for: Hetnet connectivity search provides rapid insights into how biomedical entities are related
Source: Gigascience. 2023 Jul 28;12:giad047. doi: 10.1093/gigascience/giad047 (PMC10375517; doi:10.1093/gigascience/giad047)
Supplement: giad047_Response_to_Reviewer_Comments_Original_Submission [file giad047_response_to_reviewer_comments_original_submission.pdf]

Author's Response To Reviewer Comments

Close

Our response to reviewers is available in a more formatted fashion here:
https://github.com/greenelab/connectivity-search-manuscript/blob/main/content/response-to-reviewers.md

Response to Reviewers

This document contains the response to reviewers for _GigaScience_ submission `GIGA-D-23-00005` received on 2023-02-2
Along with changes related to reviewer feedback, we [made revisions](https://github.com/greenelab/connectivity-search-manuscript/issues/42) based on editorial requests to register software applications with biotools and RRID identifiers as well
explicitly cite GitHub repositories.
To define citation metadata, we added `CITATION.cff` files to the relevant GitHub repositories.

We'd like to thank the two reviewers for their careful and insightful examination of our study.
In addition to revising the manuscript, we've responded to the points raised with additional detail here.
We are pleased that _GigaScience_ publishes reviews and responses alongside the article,
such that readers can benefit from this conversation.
This response to reviewers is [also online](https://github.com/greenelab/connectivity-search-manuscript/blob/main/content/response-to-reviewers.md) as part of the Manubot manuscript repository for this study.

Reviewer #1

> The paper is very well-written and addresses an important problem. The database appears easy to use and contains a lot o
computed data, which will be useful for researchers to query and generate useful insights. I only have a few minor comments
if addressed, could further strengthen this manuscript.

Minor comments:

> Without line and page numbers, it was a bit tricky to point out the issues.

Apologies!
The Manubot tool we're using to write the manuscript [struggles](https://github.com/manubot/rootstock/issues/244) with lin
numbering.
Some alternatives in case they're helpful.
Line numbers are available in the pre-rendered markdown source at [`05.main-text.md`](https://github.com/greenelab/connectivity-search-manuscript/blob/ebbbab424c6606a57683fa78f4c72a0bfcba01a1/content/05.main-text.md?plain=1) (replace commit hash in
with the latest `main` branch commit to see the most recent version).
Going to will redirect you to a versioned permalink of the manuscript with HTML anchors for many elements and
[Hypothesis](https://web.hypothes.is/) commenting available.
Neither of these options are as convenient as having line numbers in the submitted PDF.

> 1. "One such application" in the introduction does not read well - just "one application"

Revised.

> 2. It is nice to see that DWPCs that are not retained by the database can be generated on the fly. The para goes on to men
"while still allowing on-demand access to the full metrics for all metapaths with length ≤ 3" --- is it also possible to generate
for longer paths if needed?

The webapp is limited to displaying metapaths that are in the relational database `Metapath` table, which is currently all met

with length ≤ 3.
The backend API will respond to a query for the paths between a given source and target node across a metapath that is not
database.
For example, the following API request for a length 4 metapath succeeds:
.
While the response includes paths along with the corresponding node and edge properties, it is missing information on the D
distribution and hence the DWPC p-value.

The discussion touches on approaches future work could consider to subset all possible longer metapaths to only the most int
starting with "One solution would be to only extend metapaths detected as informative".

> 3. Below [Fig 2](https://greenelab.github.io/connectivity-search-manuscript/v/cf1000cbdfdb08a07e39cf9741d0300f76d157cf/#fig:webapp-metapaths), there is a point about the adjusted p-
see that the discussion about FDR is presented later in the manuscript (and well justified), but there could be a pointer here t
section.

We've cross-referenced the text "Bonferroni-adjusted" to the "Adjusting DWPC _p_-values" methods section.

> 4. Is there a possibility to include other computations like betweenness centrality and motifs also? This kind of data looks r
ripe for an excellent analysis of repeated motifs etc.

Existing methods for betweenness centrality and motifs are most commonly applied to homogeneous networks where there is
type of node and edge.
In the context of hetnets and this study, there are interesting parallels.
For example, given a connectivity search query of a source and target node, one could aggregate path scores by the interme
nodes, edges, or subpaths.
We explore this idea for a single metapath in the discussion [Decomposing the DWPC to assess intermediate node or edge contributions](https://doi.org/10.15363/thinklab.d228 "Daniel Himmelstein (2016) Decomposing the DWPC to assess interme
node or edge contributions. Thinklab. doi:10.15363/thinklab.d228").
The idea generalizes to aggregating paths that traverse different metapaths.
It's a good approach for reducing a large list of paths to a smaller number of intermediate nodes or edges to aid in interpreta
While not planned for the current version of the connectivity search webapp, it a promising future direction that we have add
discussion.

> 5. I found the Methods extremely long and may be a bit distracting for readers of this manuscript --- I was wondering if so
these can be moved to Supplementary.

A large portion of this study is comprised of methods development, so we are hesitant to move important details to a suppler
Our hope is that the plentiful section headings will aid readers in navigating the methods.
The revisions do include some refinements and shortening of certain methods sections.

> 6. In the section on "Details of matrix DWPC implementation", it is stated that "our matrix methods were validated". It is n
where these validations have been discussed. Supplementary?

There is no additional discussion of this validation in the manuscript.
We've updated the sentence to clarify that the validation was based on the two explicit path enumeration methods in the het
package: pure Python and Neo4j Cypher.
The validation was encoded for a smaller subset of 9 metapaths in the `hetmatpy` test suite file
[`test_path_count.py`](https://github.com/hetio/hetmatpy/blob/v0.1.0/hetmatpy/tests/test_path_count.py).

> 7. In the section on "Permuted hetnets", it is not fully clear what the parameters for XSwap algorithm was. What were the
parameters, e.g. number of swaps, etc.?

The permuted hetnets numbered 006-200 were generated in [connectivity-search-analyses#107](https://github.com/greenelab/connectivity-search-analyses/pull/107) and archived in [connectivity-search-analyses#127](https://github.com/greenelab/connectivity-search-analyses/pull/127).
We retained the permutation progress statistics in [`permutations/stats.tsv`](https://github.com/greenelab/connectivity-sea

analyses/blob/15f1925c0481d8e6bab8b0931f48f2fad388c68c/data/hetionet-v1.0.hetmat/permutations/stats.tsv).
Looking at this table, we can confirm that we applied the default XSwap parameters from the `hetnetpy.permute.permute_gr
[function](https://github.com/hetio/hetnetpy/blob/v0.3.1/hetnetpy/permute.py#L8), which sets `multiplier=10`,
meaning that for each metaedge, we attempted 10 times the number of swaps as actual edges.
This default multiplier of 10 was initially introduced in [Himmelstein & Baranzini (2015)](https://doi.org/10.1371/journal.pcbi.1004259).
Additional [investigation](https://doi.org/10.15363/thinklab.d178) in Project Rephetio found that a multiplier of 4 provided su
randomization.
While Project Rephetio adopted a multiplier of 4, which applies to permuted hetnets 001-005 (see Rephetio
[stats.tsv](`https://github.com/dhimmel/integrate/blob/ebd71cd2157d26e52646b5b483f5c70293a84f71/data/permuted/stat
for permutations 006-200 generated by this study we used the larger default multiplier 10 to increase the margin of safety.

> 8. In the section on "[Details of the gamma-hurdle distribution](https://greenelab.github.io/connectivity-search-manuscript/v/cf1000cbdfdb08a07e39cf9741d0300f76d157cf/#details-of-the-gamma-hurdle-distribution)", there is perhaps a
equation below the second statement of "The probability of a draw from the distribution is"

We updated the wording to be "The gamma-hurdle distribution is defined over the support [0, ∞). The probability of a draw,
the gamma-hurdle distribution is given as follows:".
Since the distribution is defined only from zero (inclusive) to positive infinity, the two lines of equations that follow fully defin
distribution.

> 9. The validation here which points to an
[ipynb](https://nbviewer.org/github/greenelab/hetmech/blob/025bdf8d5e63725ca2482d61fd8e421bf0001f93/explore/gamm
hurdle/parameter_estimates.ipynb), could be put in Supplement.

We replaced the [nbviewer
link](https://nbviewer.org/github/greenelab/hetmech/blob/025bdf8d5e63725ca2482d61fd8e421bf0001f93/explore/gamma-hurdle/parameter_estimates.ipynb) with a direct [GitHub link](https://github.com/greenelab/connectivity-search-analyses/blob/1c6827ce2544c17cef42bbccf098a312f2c44f97/explore/gamma-hurdle/parameter_estimates.ipynb), since GitH
now fully capable of rendering this `.ipynb` file. This removes the dependency on `nbviewer.org` as a viewer of "supplemen
project content.

> 10. In the section on "[Prioritizing enriched metapaths for database storage](https://greenelab.github.io/connectivity-searc
manuscript/v/cf1000cbdfdb08a07e39cf9741d0300f76d157cf/#prioritizing-enriched-metapaths-for-database-storage)", what
logic underlying the choice of parameters? "For metapaths with length ≥ 2, we chose an adjusted pvalue threshold of 5 × (ns
ntarget)^−0.3".

The objectives and effect of the parameters are described in the manuscript.
As for setting their specific values, the multiplier of 5 was chosen to "saturate the available database capacity".
Setting the exponent at −0.3 involved a bit of guesswork, which was prototyped in the [`metapath-thresholds.ipynb`](https://github.com/greenelab/connectivity-search-analyses/blob/1c6827ce2544c17cef42bbccf098a312f2c44f97/explore/metapath-thresholds/metapath-thresholds.ipynb) noteb
We manually evaluated several exponent choices and reviewed the resulting p-value thresholds,
looking for a threshold to ensure that the database capacity was not overwhelmingly devoted to large-sized DWPC matrices w
providing the ability for highly significant metapaths for large-sized DWPC matrices to be retained.
The −0.3 exponent achieved these objectives, so we adopted it as a way to optimize the storage capacity towards presumed
interest.

> 11. Under "Visual Design", are the colours chosen "colour-blind friendly"?

Good question.
Using the [hclwizard CVD emulator](http://hclwizard.org:3000/cvdemulator/), we've
[simulated](https://github.com/hetio/hetionet/pull/18#issuecomment-1452877325) the new and old Hetio color palettes for
CVDs at 100% severity:

![hetionet metagraph colorblindness simulator](https://user-images.githubusercontent.com/1117703/222621153-a0954237-
4729-a2f2-9d46521f96fb.png)

Some colors are harder to differentiate. It's worth noting that we often show metanode abbreviations or node names along w
colors,
such that color is helpful but not essential for usage and interpretation.

Reviewer #2:

> In this work Himmelstein and collaborators introduce a statistically controlled way of extracting significant node pairs in
heterogeneous networks (hetnets) without relying on a ground truth and related training. The method "explains" why two no
significantly connected by extracting the metapaths most responsible for the enrichment. This is based on computing a null
distribution of the DWPC, which allows assigning a P-value to each metapath joining two nodes, and then to visualize the indi
paths responsible for the enrichment. The method is novel and significant, and can be in principle be applied to many hetnets
sciences and beyond, when a ground truth is not available or not desirable as it would introduce bias. The software tools dev
appear to be readily available to other researchers.

Major comment:

> If I understand correctly, given two nodes (say "Alzheimer disease" and "Circadian rhythm") the method extracts, in a stat
controlled way, the most significant metapaths joining the two nodes, and then the individual paths responsible for the enrich
But this is not the most obvious question a life scientist would ask the network, which would be instead something like "Whic
pathways most significantly connected to "Alzheimer disease"? Indeed this type of question would be the one to ask when ai
drug repurposing (possibly replacing "pathways" with "compounds" or "pharmacologic classes"). Based on Fig. 4A, the pathw
presented, or "suggested," in decreasing order of number of metapaths, but this is hardly a ranking by significance. Would it
possible to summarize the results in such a way as to rank the pathway nodes connected to a given disease node by significa
more generally to rank the nodes of a certain type by the significance of their connection to a given node of another type)? T
should be discussed.

The reviewer's understanding is correct.
The connectivity search webpage takes a metapath/path based approach to communicating query results.
Many users will also be interested in metanode/node based results.
The discussion now notes the possibility of "aggregating path scores across intermediate nodes or edges".
We agree with the reviewer that this enhancement to the webapp would bring added utility and is an excellent future directio
Furthermore, we now summarize the MechRepoNet/DrugMechDB [study](http://doi.org/10.1093/bioinformatics/btac205) in t
introduction, which performed node aggregation techniques, such that readers can further appreciate this possibility.

Minor comments

> I also have several minor concerns.
>
> (1) The authors introduce and compute a null distribution of the DWPC which takes into account node degree in a statistica
controlled way when evaluating the connectivity between two nodes. However, the DWPC itself does take into account node
as the name implies, and contains a tunable parameter that can be optimized, at least when a ground truth is available (as i
39](https://greenelab.github.io/connectivity-search-manuscript/v/cf1000cbdfdb08a07e39cf9741d0300f76d157cf/#ref-WkPlH
"Reference entry for Heterogeneous Network Edge Prediction: A Data Integration Approach to Prioritize Disease-Associated G
by the same first author). I understand such tuning is not possible when, as in the present case, no ground truth is available,
authors should make this point more clearly.

The DWPC metric itself does have a damping parameter to penalize paths that traverse high degree nodes.
However, the degree-weighting of paths does not fully correct for source and target degree as seen in the figure "Path-based
vary by node degree and network permutation status".
Specifically, the gamma-hurdle model for the null DWPC distribution is able to correct for two additional aspects of DWPCs:

1. The proportion of DWPCs that are zero (i.e. no paths occurred) varies by source and target degree as shown in the "% Nor
DWPCs" panel.
2. For nonzero DWPCs, degree-weighting of paths still does not fully correct for source and target degree as shown in the "Me
Nonzero DWPC" panel.

Note that the degree-weighting of paths is still important even when constructing a null distribution like we do here.
That is because our null distributions are based only on source and target node degrees and cannot penalize high degree inte[illegible] nodes that are traversed by specific paths.

> (2) I find [Fig. 1B](https://greenelab.github.io/connectivity-search-manuscript/v/cf1000cbdfdb08a07e39cf9741d0300f76d157cf/#fig:rephetio) a bit confusing: according to the legend, the top [illegible] known treatments, which should have higher than expected connectivity. However, based on the colors as explained by the l[illegible] the bottom treatment/disease pairs seem to have higher connectivity

The caption had top/bottom reversed!
Many thanks for this keen observation.

> (3) The acronym DWPC is defined after it has been used several times

We now use the long form before the first occurrence of DWPC.

> (4) The legend of [Figure 2](https://greenelab.github.io/connectivity-search-manuscript/v/cf1000cbdfdb08a07e39cf9741d0300f76d157cf/#fig:webapp-metapaths) should specify that these results apply [illegible] nodes "Alzheimer disease" and "Circadian rhythm", although this becomes clear in [Fig. 4](https://greenelab.github.io/conne[illegible] search-manuscript/v/cf1000cbdfdb08a07e39cf9741d0300f76d157cf/#fig:webapp)

We've updated the figure caption.

> (5) I don't think [Figure 3](https://greenelab.github.io/connectivity-search-manuscript/v/cf1000cbdfdb08a07e39cf9741d0300f76d157cf/#fig:website), representing the home page of the web site, is es[illegible] useful

We moved the figure titled "Homepage of the Hetio website" to the **Methods > Frontend > Hetio Website** section,
so it no longer interrupts the more essential flow of figures in Results.
We decided to retain the figure because it demonstrates aspects of the website revamping that occurred as part of this study[illegible]
As a result, figure numbers have changed in the revised manuscript.

> (6) I found [Fig. 4](https://greenelab.github.io/connectivity-search-manuscript/v/cf1000cbdfdb08a07e39cf9741d0300f76d157cf/#fig:webapp) confusing: the sum of the path counts for the sele[illegible] metapaths in panel B is way larger than the 425 results shown in Panel C. As far as I understand no path can belong to more [illegible] one metapaths, so is there some further selection here?

The API `paths` endpoint sets a default `limit=100` meaning that at most 100 paths are returned for a given (metapath, so[illegible] node, target node) triplet ([source code](https://github.com/greenelab/connectivity-search-backend/blob/0a9a5a694c1df6a3a62d75d8b1122218f8bfb60f/dj_hetmech_app/views.py#L217-L234)).
While the API will accept other values for `limit`, the webapp does not expose this parameter.
We've found interactive web-based users rarely benefit from seeing more than 100 paths for a selected metapath.
Furthermore, some limit is necessary as excessively large path counts would bog down the webapp.

In a live session of panel B via the webapp, when the user hovers over the show _DaGiGpPW_ metapath checkbox, the documentation tooltip [notes](https://github.com/greenelab/connectivity-search-frontend/blob/8fae3b0c61ef7d831414b3d964de4d0120988ce2/src/metapath-results/table.js#L349-L354) "Show the top 100 [illegible] 479 paths in the paths table". We've updated the figure caption to note this limit.

> (7) The "[Frontend](https://greenelab.github.io/connectivity-search-manuscript/v/cf1000cbdfdb08a07e39cf9741d0300f76d157cf/#frontend)" section of the Methods seems a bit too detailed for [illegible] Gigascience audience

We shortened the frontend section, while still retaining detail about the implementation and decision making process that mig[illegible] interest to readers looking to produce similar style webapps.
Since this study contained considerable web development efforts, we would like the manuscript to dutifully reflect this work.

Close
