# Supplementary material for: Hetnet connectivity search provides rapid insights into how biomedical entities are related
Source: Gigascience. 2023 Jul 28;12:giad047. doi: 10.1093/gigascience/giad047 (PMC10375517; doi:10.1093/gigascience/giad047)
Supplement: giad047_Response_to_Reviewer_Comments_Revision_1 [file giad047_response_to_reviewer_comments_revision_1.pdf]

Author's Response To Reviewer Comments

Close

We made the following changes:

* added keywords
* frontmatter: remove author funding, add emails
* copy in gigascience style references, in addition to the manubot style references

Please note, hyperlinks can be dropped if necessary, we have decided not to create any more references for them. The DOCX has the references in two styles, first the gigascience style according to your CSL (https://github.com/citation-style-language/styles/blob/256250be9c6ab34bba4a070b5c050f7848b37beb/gigascience.csl) and then the Manubot style which in some cases appears to provide more pertinent information. You can reference the HTML (https://greenelab.github.io/connectivity-search-manuscript/v/b6532fafbc126c0818c612febf86968e6fa8e21b/) or PDF (https://greenelab.github.io/connectivity-search-manuscript/v/b6532fafbc126c0818c612febf86968e6fa8e21b/manuscript.pdf) manuscript if there are questions about the DOCX formatting.

Close
