# Supplementary material for: Hetnet connectivity search provides rapid insights into how biomedical entities are related
Source: Gigascience. 2023 Jul 28;12:giad047. doi: 10.1093/gigascience/giad047 (PMC10375517; doi:10.1093/gigascience/giad047)
Supplement: giad047_Response_to_Reviewer_Comments_Revision_2 [file giad047_response_to_reviewer_comments_revision_2.pdf]

Author's Response To Reviewer Comments

Close

We've removed all main text hyperlinks. The remaining main text links are internal to the manuscript (i.e. to figures, sections, tables, references).

We have added the DOI to the GigaDB reference by tweaking the CSL style for Gigascience. We provide the references in two formats: first the Gigascience format as per the CSL and second the Manubot style. If the references need any more reformatting, it is our preference that this be handled by the production team.

We have registered RRID:SCR_023630 and included it in the manuscript.

Close
