# Supplementary material for: Hetnet connectivity search provides rapid insights into how biomedical entities are related
Source: Gigascience. 2023 Jul 28;12:giad047. doi: 10.1093/gigascience/giad047 (PMC10375517; doi:10.1093/gigascience/giad047)
Supplement: giad047_Reviewer_1_Report_Original_Submission — Karthik Raman -- 2/8/2023 Reviewed [file giad047_reviewer_1_report_original_submission.pdf]

Reviewer Report

Title: Hetnet connectivity search provides rapid insights into how two biomedical entities are related

Version: Original Submission **Date:** 2/8/2023

Reviewer name: Karthik Raman

Reviewer Comments to Author:

The paper is very well-written and addresses an important problem. The database appears easy to use and contains a lot of pre-computed data, which will be useful for researchers to query and generate useful insights. I only have a few minor comments, which if addressed, could further strengthen this manuscript.Minor comments:Without line and page numbers, it was a bit tricky to point out the issues.1. "One such application" in the introduction does not read well - just "one application"2. It is nice to see that DWPCs that are not retained by the database can be generated on the fly. The para goes on to mention "while still allowing on-demand access to the full metrics for all metapaths with length â‰¤ 3" --- is it also possible to generate metrics for longer paths if needed?3. Below Fig 2, there is a point about the adjusted p-value. I see that the discussion about FDR is presented later in the manuscript (and well justified), but there could be a pointer here to that section.4. Is there a possibility to include other computations like betweenness centrality and motifs also? This kind of data looks really ripe for an excellent analysis of repeated motifs etc.5. I found the Methods extremely long and may be a bit distracting for readers of this manuscript --- I was wondering if some of these can be moved to Supplementary.6. In the section on "Details of matrix DWPC implementation", it is stated that "our matrix methods were validated". It is not clear where these validations have been discussed. Supplementary?7. In the section on "Permuted hetnets", it is not fully clear what the parameters for XSwap algorithm was. What were the parameters, e.g. number of swaps, etc.?8. In the section on "Details of the gamma-hurdle distribution", there is perhaps a missing equation below the second statement of "The probability of a draw from the distribution is"9. The validation here which points to an ipynb, could be put in Supplement.10. In the section on "Prioritizing enriched metapaths for database storage", what is the logic underlying the choice of parameters? "For metapaths with length â‰¥ 2, we chose an adjusted pvalue threshold of 5 Ã— (nsource Ã— ntarget)^âˆ’0.3".11. Under "Visual Design", are the colours chosen "colour-blind friendly"?

Level of Interest

Please indicate how interesting you found the manuscript: Choose an item.

Quality of Written English

Please indicate the quality of language in the manuscript: Choose an item.

Declaration of Competing Interests

Please complete a declaration of competing interests, considering the following questions:

- Have you in the past five years received reimbursements, fees, funding, or salary from an organisation that may in any way gain or lose financially from the publication of this manuscript, either now or in the future?
- Do you hold any stocks or shares in an organisation that may in any way gain or lose financially from the publication of this manuscript, either now or in the future?
- Do you hold or are you currently applying for any patents relating to the content of the manuscript?
- Have you received reimbursements, fees, funding, or salary from an organization that holds or has applied for patents relating to the content of the manuscript?
- Do you have any other financial competing interests?
- Do you have any non-financial competing interests in relation to this paper?

If you can answer no to all of the above, write 'I declare that I have no competing interests' below. If your reply is yes to any, please give details below.

I declare that I have no competing interests

I agree to the open peer review policy of the journal. I understand that my name will be included on my report to the authors and, if the manuscript is accepted for publication, my named report including any attachments I upload will be posted on the website along with the authors' responses. I agree for my report to be made available under an Open Access Creative Commons CC-BY license (http://creativecommons.org/licenses/by/4.0/). I understand that any comments which I do not wish to be included in my named report can be included as confidential comments to the editors, which will not be published.

Choose an item.

To further support our reviewers, we have joined with Publons, where you can gain additional credit to further highlight your hard work (see: https://publons.com/journal/530/gigascience). On publication of this paper, your review will be automatically added to Publons, you can then choose whether or not to claim your Publons credit. I understand this statement.

Yes Choose an item.
