# Supplementary material for: Hetnet connectivity search provides rapid insights into how biomedical entities are related
Source: Gigascience. 2023 Jul 28;12:giad047. doi: 10.1093/gigascience/giad047 (PMC10375517; doi:10.1093/gigascience/giad047)
Supplement: giad047_Reviewer_2_Report_Original_Submission — Paolo Provero -- 2/13/2023 Reviewed [file giad047_reviewer_2_report_original_submission.pdf]

Reviewer Report

Title: Hetnet connectivity search provides rapid insights into how two biomedical entities are related

Version: Original Submission **Date:** 2/13/2023

Reviewer name: Paolo Provero

Reviewer Comments to Author:

In this work Himmelstein and collaborators introduce a statistically controlled way of extracting significant node pairs in heterogeneous networks (hetnets) without relying on a ground truth and related training. The method "explains" why two nodes are significantly connected by extracting the metapaths most responsible for the enrichment. This is based on computing a null distribution of the DWPC, which allows assigning a P-value to each metapath joining two nodes, and then to visualize the individual paths responsible for the enrichment. The method is novel and significant, and can be in principle be applied to many hetnets, in life sciences and beyond, when a ground truth is not available or not desirable as it would introduce bias. The software tools developed appear to be readily available to other researchers.Major comment:If I understand correctly, given two nodes (say "Alzheimer disease" and "Circadian rhythm") the method extracts, in a statistically controlled way, the most significant metapaths joining the two nodes, and then the individual paths responsible for the enrichment. But this is not the most obvious question a life scientist would ask the network, which would be instead something like "Which are the pathways most significantly connected to "Alzheimer disease"? Indeed this type of question would be the one to ask when aiming for drug repurposing (possibly replacing "pathways" with "compounds" or "pharmacologic classes"). Based on Fig. 4A, the pathways are presented, or "suggested," in decreasing order of number of metapaths, but this is hardly a ranking by significance. Would it be possible to summarize the results in such a way as to rank the pathway nodes connected to a given disease node by significance (or more generally to rank the nodes of a certain type by the significance of their connection to a given node of another type)? This should be discussed.I also have several minor concerns.(1) The authors introduce and compute a null distribution of the DWPC which takes into account node degree in a statistically controlled way when evaluating the connectivity between two nodes. However, the DWPC itself does take into account node degree, as the name implies, and contains a tunable parameter that can be optimized, at least when a ground truth is available (as in Ref 39 by the same first author). I understand such tuning is not possible when, as in the present case, no ground truth is available, but the authors should make this point more clearly.(2) I find Fig. 1B a bit confusing: according to the legend, the top rows are known treatments, which should have higher than expected connectivity. However, based on the colors as explained by the legend, the bottom treatment/disease pairs seem to have higher connectivity(3) The acronym DWPC is defined after it has been used several times(4) The legend of Figure 2 should specify that these results apply to the nodes "Alzheimer disease" and "Circadian rhythm", although this becomes clear in Fig. 4(5) I don't think Figure 3, representing the home page of the web site, is especially useful(6) I found Fig. 4 confusing: the sum of the path counts for the selected metapaths in panel B is way larger than the 425 results shown in Panel C. As far as I understand no path can belong to more than one metapaths, so is there some further

selection here?(7) The "Frontend" section of the Methods seems a bit too detailed for the Gigascience audience

Level of Interest

Please indicate how interesting you found the manuscript: Choose an item.

Quality of Written English

Please indicate the quality of language in the manuscript: Choose an item.

Declaration of Competing Interests

Please complete a declaration of competing interests, considering the following questions:

- Have you in the past five years received reimbursements, fees, funding, or salary from an organisation that may in any way gain or lose financially from the publication of this manuscript, either now or in the future?
- Do you hold any stocks or shares in an organisation that may in any way gain or lose financially from the publication of this manuscript, either now or in the future?
- Do you hold or are you currently applying for any patents relating to the content of the manuscript?
- Have you received reimbursements, fees, funding, or salary from an organization that holds or has applied for patents relating to the content of the manuscript?
- Do you have any other financial competing interests?
- Do you have any non-financial competing interests in relation to this paper?

If you can answer no to all of the above, write 'I declare that I have no competing interests' below. If your reply is yes to any, please give details below.

I declare that I have no competing interests

I agree to the open peer review policy of the journal. I understand that my name will be included on my report to the authors and, if the manuscript is accepted for publication, my named report including any attachments I upload will be posted on the website along with the authors' responses. I agree for my report to be made available under an Open Access Creative Commons CC-BY license (http://creativecommons.org/licenses/by/4.0/). I understand that any comments which I do not wish to be included in my named report can be included as confidential comments to the editors, which will not be published.

Choose an item.

To further support our reviewers, we have joined with Publons, where you can gain additional credit to further highlight your hard work (see: https://publons.com/journal/530/gigascience). On publication of this paper, your review will be automatically added to Publons, you can then choose whether or not to claim your Publons credit. I understand this statement.

Yes Choose an item.
